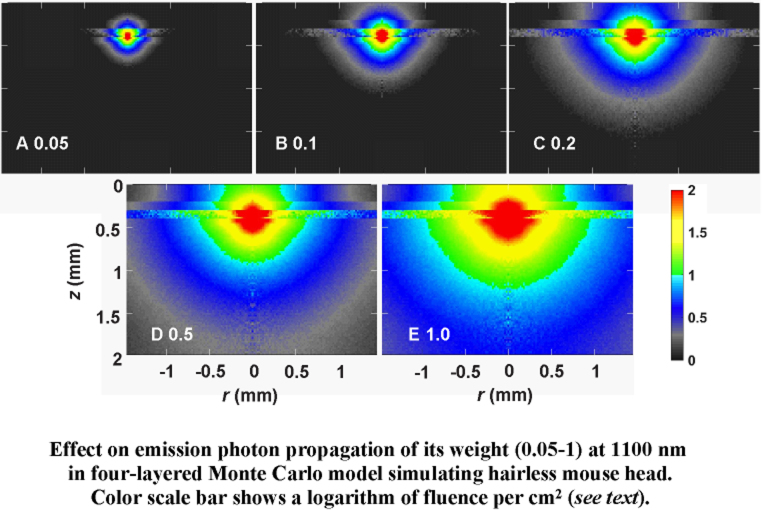# Optimal focus evaluated using Monte Carlo simulation in non-invasive neuroimaging in the second near-infrared window

**DOI:** 10.1016/j.mex.2019.09.010

**Published:** 2019-09-12

**Authors:** Tatsuto Iida, Hiro Yamato, Takashi Jin, Yasutomo Nomura

**Affiliations:** a Department of Systems Life Engineering, Maebashi Institute of Technology, Maebashi, 371-0816, Japan; b Laboratory for Nano-Bio Probes, RIKEN Center for Biosystems Dynamics Research, Suita, 565-0874, Japan

**Keywords:** Focusing on cerebrovascular structures behind intact scalp using second near-infrared fluorescence, Second optical window, Angiography, Mouse, Epifluorescence macro zoom microscope, Quantum dots, InGaAs camera

## Abstract

Adjusting the focal plane through the intact scalp of mice is crucial in novel angiography of cerebral vasculature using quantum dots emitting second near-infrared light at a wavelength of 1100 nm. Reagents were administered through the caudal vein. When we focused 0.4 mm below the scalp surface, based on the anatomical properties of mice reported previously, the intensity of clear fluorescence images observed transiently under a microscope became very weak within several seconds. The remaining time was extremely short to repeat adjustment of the focal plane. To investigate focus, photons exciting quantum dots at depths of 0.4, 0.8, 1.4, and 2.0 mm and emission photons were tracked in a four-layered Monte Carlo model including the scalp, skull, cerebrospinal fluid, and cortex. Based on the most near-ballistic photons emitted from quantum dots at 0.4 mm depth and specification of the microscope used, including numerical aperture and depth of field, the optimal focus plane was set.

• Novel angiography for cerebrovascular structures was proposed using quantum dots with second near-infrared fluorescence.
• Anatomical properties reported previously allowed focusing 0.4 mm below the surface of intact scalp before observation under fluorescence.
• Clear images of cerebrovascular structures were attributed to many near-ballistic photons emitted from quantum dots at 0.4 mm depth.

## Graphical abstract

**Figure:**
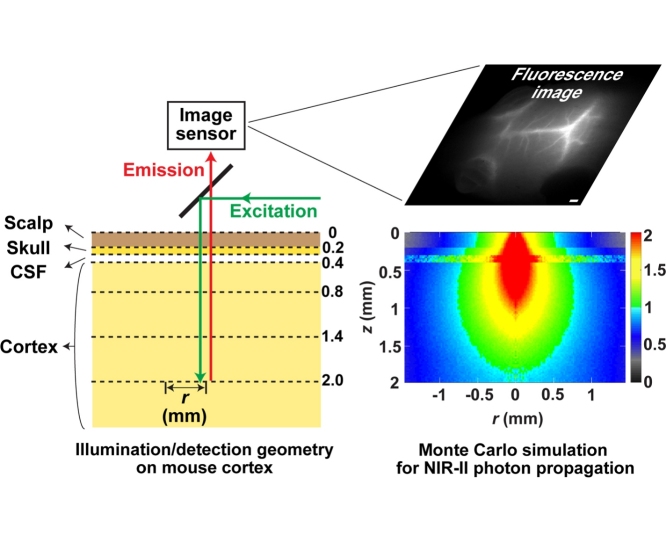

**Specification Table**

**Table:** 

Subject area:	Neuroscience
More specific subject area:	In vivo neuroimaging
Method name:	Focusing on cerebrovascular structures behind intact scalp using second near-infrared fluorescence
Name and reference of original method:	Not applicable
Resource availability:	hairless mice Hos:HR-1 from Hoshino Laboratory Animals of Japan, macro-zoom fluorescence microscope, InGaAs camera

## Method details

### Fluorescence imaging system in the second near-infrared window

The surface of intact scalp from the parietal to frontal area was illuminated using the laser diode BWF1(B&W Tek, USA) that emitted light at a wavelength of 785 nm. The maximum excitation power on the sample stage was 25.5 mW/cm^2^. A research MVX-10 macro-zoom fluorescence microscope (Olympus, Japan) coupled with the C10633-34 InGaAs camera (Hamamatsu photonics, Japan) was used for the imaging. A filter set comprising an excitation filter for a 785 nm laser, dichroic mirror to reflect the 785 nm laser and transmit over a wavelength of 800 nm, and a long-pass emission filter with a cut-off wavelength of 800 nm was used. Furthermore, an emission filter with a wavelength of 1100 ± 25 nm was used and placed after the filter set. Camera control and image acquisition (exposure time: 100 ms/frame) were performed using HCImage (Hamamatsu photonics, Japan) [1]. In the present study, we prepared and used the highly fluorescent PbS/CdS core-shell quantum dots QD1100 conjugated with bovine serum albumin (emission peak: 1100 nm, quantum yields: 17% in water) [2].

### Animal procedures

The experimental procedures in the present study were approved by the Animal Care and Use Committee of the Maebashi Institute of Technology (AN-18-006). Data were obtained from 4-5week-old hairless male Hos:HR-1 mice (Hoshino Laboratory Animals, Japan). The animals were housed in cages with ad libitum access to food pellets and water. They were maintained under a 12-h light/dark cycle. The Hos:HR-1 strain loses its hair coat rapidly and completely due to an abnormal second hair cycle starting at 2 weeks of age, thereby becoming completely hairless [3]. Recently, in non-invasive transcranial optical vascular imaging (nTOVI), young mice with heads treated with hair removal cream for shaving were used [4]. In the present study, we used hairless mice without the need for hair removal just before imaging, which had no effect on brain temperature during the procedure. Each animal was anesthetized and maintained with 1.5–2.0% isoflurane (v/v). The animals were placed in a special holder equipped with a warm plate which constantly maintained body temperature at 37 °C using the ATC-402 temperature control system (Unique Medical, Japan).

### Fluorescence imaging of cerebrovascular structure

**Fig. 1:**
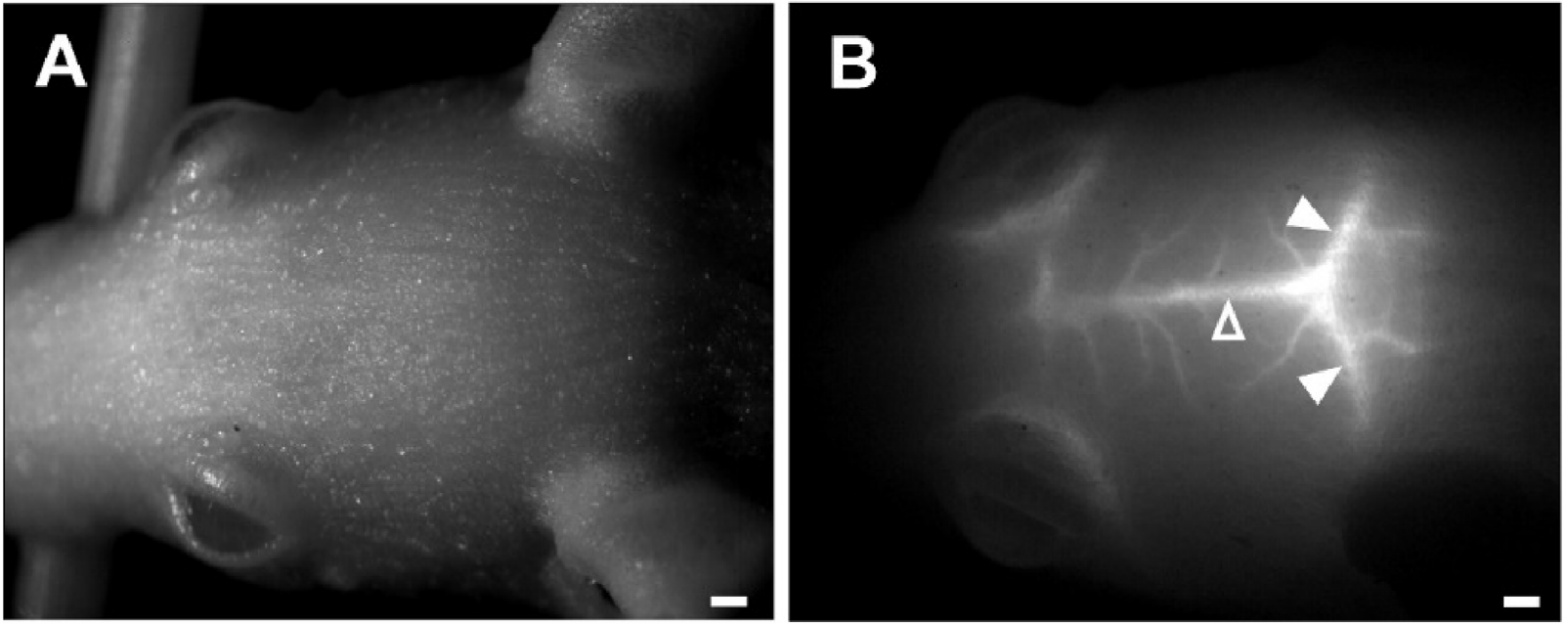
**NIR-II fluorescence image of cerebral blood vessels in a mouse.** (A) Bright field, (B) fluorescence image focusing 0.4 mm below the surface of the intact scalp (scale bar =1 mm). Open arrowhead: superior sagittal sinus, closed arrowhead: transverse sinus.

Several seconds after bolus injection of 50 μL of 1 μM QD1100 into the caudal vein of a mouse, weak fluorescence near the middle cerebral arteries appeared occasionally. Then, strong fluorescence in the venous phase masked that in the weak arterial phase. As shown with arrowheads in Fig. 1, the left and right transverse and sagittal sinuses were observed when we focused 0.4 mm below the scalp surface based on the anatomical properties of mice reported previously [5]. Subsequently, fluorescence signals gradually weakened. In contrast to nTOVI, a cerebrovascular structure appeared without hair removal just before imaging [4]. Furthermore, the authors treated the scalp with a mixture of glycerol and liquid paraffin oil, which was not required in the present study. Although we obtained similar images in nude mice, it was necessary to shave and treat the scalp of BALB mice with the translucent oil. These differences may be attributable to not only the use of hairless mice but also QD1100. As described later in the section of Monte Carlo simulation, photons emitted at a wavelength of 1100 nm in the second near-infrared window (NIR-II) penetrate tissues more deeply than those emitted in the conventional near-infrared region (NIR-I) as used in nTOVI. Because we could not directly observe blood vessels without fluorescence, it was difficult to focus on them previously. Although the lack of an indicator such as fluorescence was occasionally implemented by digital image processing or diaphragm tuning, these efforts were not very effective in the present study. Next, by focusing at a depth of 0.4 mm below the sculp surface, examination was conducted using Monte Carlo simulation because the photon propagation of excitation and emission was dependent on image quality.

### Four-layered Monte Carlo model

The four-layered Monte Carlo model was developed based on the three-layered model [5]. The software for the fluorescence Monte Carlo model was developed using C programming language with Microsoft Visual Studio. The model was essentially based on the concepts outlined by Welch et al. [6]. In Monte Carlo simulation, the model comprised three parts. The first part was the simulation of photon propagation at excitation wavelength (785 nm) with the corresponding optical properties, based on the computation routines reported by Wang et al. [7]. The weight of excitation photons decreased at each scattering and absorption interaction site. In the second part, weight was inherited by fluorescence depending on photon number. Lastly, the third part was the simulation of photon propagation at emission wavelength (1100 nm). The geometry of the model is outlined in the Graphical abstract. Although attention was needed for imaging the non-flat surface, a point source was located at the plane surface of the turbid medium, namely scalp on the skull. Photons were collected at a light axis-detector separation *r*. Each layer was infinitely wide, and the thickness of the scalp, skull, and cerebrospinal fluid (CSF) was set to 0.2, 0.1, and 0.1 mm, respectively, based on the anatomical properties of the mouse. Except the scattering coefficient *μ_s_* and anisotropy *g* of CSF, optical parameters to simulate the observation space of mouse head were found in literature. Because CSF is a clear solution, *μ_s_* and *g* was set to 0 and 1, respectively, according to their definitions. Scalp was regarded as skin. As shown in Table 1, each layer had optical parameters specific for tissues at specific wavelengths.

**Table 1:** Optical parameters for each tissue. Regardless of the wavelength, the anisotropy *g* of skull and cortex was assumed to be 0.9, and the *g* of CSF and air to be 1. Values for each parameter were set according to previous studies [[8], [9], [10], [11], [12]].

Wavelength (nm)	Tissue	*n*	*μ_a_* (cm^−1^)	*μ_s_* (cm^−1^)	*g*	Thickness (mm)
785	Skin	1.37	1.6	238.9	0.9	0.2
	Skull	1.454	0.087	162.7	0.9	0.1
	CSF	1.33	0.1	0	1	0.1
	Cortex	1.368	0.087	76.2	0.9	0, 0.4, 1, and 1.6

1100	Air	1	0.001	0	1	0.1
	Skin	1.37	0.35	171.5	0.9	0.2
	Skull	1.45	0.4	135.6	0.9	0.1
	CSF	1.33	1	0	1	0.1
	Cortex	1.368	0.5	71.4	0.9	0, 0.4, 1, and 1.6

An infinitely narrow beam comprising 10^6^ photons entered from the skin surface. The path length to a photon-tissue interaction was calculated based on random numbers and total attenuation coefficient: *μ_t_* = *μ_s_* + *μ_a_*. Each photon had an initial weight corresponding to 1. As photons reached an interaction site, they lost *μ_a_*/*μ_t_* of their weight. Toward the next interaction site, an isotropic azimuth and a zenith angle with highly forward scattering property were generated using different random numbers. When photons crossed the boundary between two layers, they were reflected or refracted according to the Fresnel reflectance. When light of an intensity *I*_0_ (i.e., photon weight) crosses the boundary from a medium with refractive index *n*_1_ to that with refractive index *n*_2_, intensity decreases to *I*,

(1) $$I=I_{0}{\left(\frac{n_{1}-n_{2}}{n_{1}+n_{2}}\right)}^{2},$$

When incident angle *α* is larger than critical angle *θ_c_*, light is reflected at emergent angle *α*.

(2) $$\theta_{c}=sin^{-1}\left(n_{2}/n_{1}\right),$$

When incident angle *α* is smaller than critical angle *θ_c_*, light is refracted at refraction angle *β*.

(3) $$\beta =sin^{-1}\left(\frac{n_{1}}{n_{2}}\sin\alpha \right),$$

Quantum dots were excited and emitted when photons entering from the skin reached them. Subsequently, they became a new light source that emitted isotropic fluorescence depending on the sum of excitation photon weights at a specific depth. Near-axis coaxial fluorescence photons were captured by an image sensor using an optical system of our epifluorescence macro zoom microscope (see Graphical abstract).

### Propagation of NIR-1 and NIR-II light

**Fig. 2:**
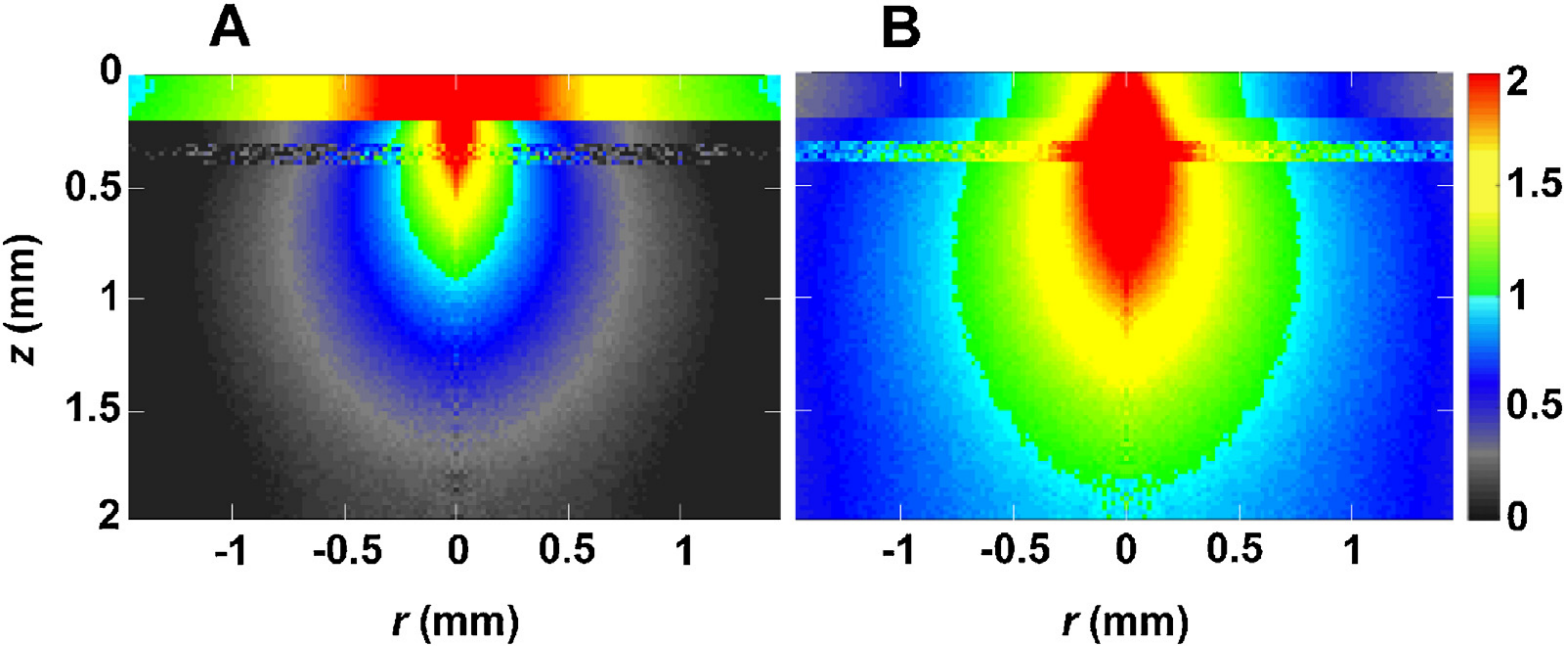
**Light propagation in the four-layered Monte Carlo model simulating hairless mice.** (A) 785 nm in NIR-I and, (B) 1100 nm in NIR-II. The depth from the scalp surface (0 mm) is expressed on the z scale (mm). The r-scale is the distance from the light axis (0 mm). The color scale bar denotes a logarithm of fluence per cm^2^.

First, we compared the differences in photon propagation between NIR-I and NIR-II using the four-layered Monte Carlo model, which simulated a hairless mouse head including the scalp, skull, CSF, and cortex. When photons entered the model with a strong forward scattering property, most photons propagated along and from the light axis, and partially lost weight due to their interaction with tissue of scattering and absorption. Fig. 2 shows photon propagation as internal photon distribution based on photon fluence, which scores absorbed photon weight in grid elements (*r*, *z*). Here, fluence is a quantity converted per photon and per cm^2^. In the skin, the absorption coefficient at 785 nm in NIR-I was 4-fold higher than that at 1100 nm in NIR-II. In addition, the scattering coefficient at 785 nm was 1.4-fold higher than that at 1100 nm. Thus, the spatial distribution of photons in NIR-I in the skin spread wider than that in NIR-II and the strong absorption made the area with high photon fluence wider than NIR-II. Near-axis NIR-II photons transmitted though the skin were considered more in number than NIR-I photons and to have more weight than NIR-I photons. On the other hand, in the skull, the absorption coefficient at 1100 nm in NIR-II was 4-fold higher than that at 785 nm in NIR-I although the scattering coefficient in NIR-II was 83% of that in NIR-I. This resulted in a high photon fluence area in NIR-II wider than that in NIR-I. The results for transverse propagation within CSF were similar to those of goniometric measurement for estimating anisotropy *g* [8]. In the measurement examining the dependency of scattering angle on light intensity from very thin tissue samples, the total internal reflection at the glass/saline interface prevented light from exiting the tissue and reaching the detector at such angles. Transverse propagation within the CSF layer in NIR-II was remarkably wider than that in NIR-I. In this model, the cortex was thicker than the skin and skull. Because the scattering coefficient of the cortex was approximately half of that of the skin and skull, the photons in the cortex penetrated deeper. The high fluence area was possibly wider in NIR-II than in NIR-I because the absorption coefficient of NIR-II is 5-fold higher than that of NIR-I. From the viewpoint of photon propagation, NIR-II appears to be suitable for non-invasive neuroimaging in mice. Considering the mechanism underlying fluorescence, we examined the effect of initial photon weight (0.05–1) on photon propagation of excitation at a wavelength of 785 nm (Fig. S1) and emission at a wavelength of 1100 nm (Fig. S2) in the four-layered Monte Carlo model with QD1100 localized at ***z*** = 0.4 mm and ***r*** =0 mm. Excitation photons propagated deeper when the excitation light became stronger, i.e., photon weight increased. In contrast, emission photons propagated more wide because of isotropic emission from quantum dots.

### NIR-II fluorescence photon propagation from a different depth

**Fig. 3:**
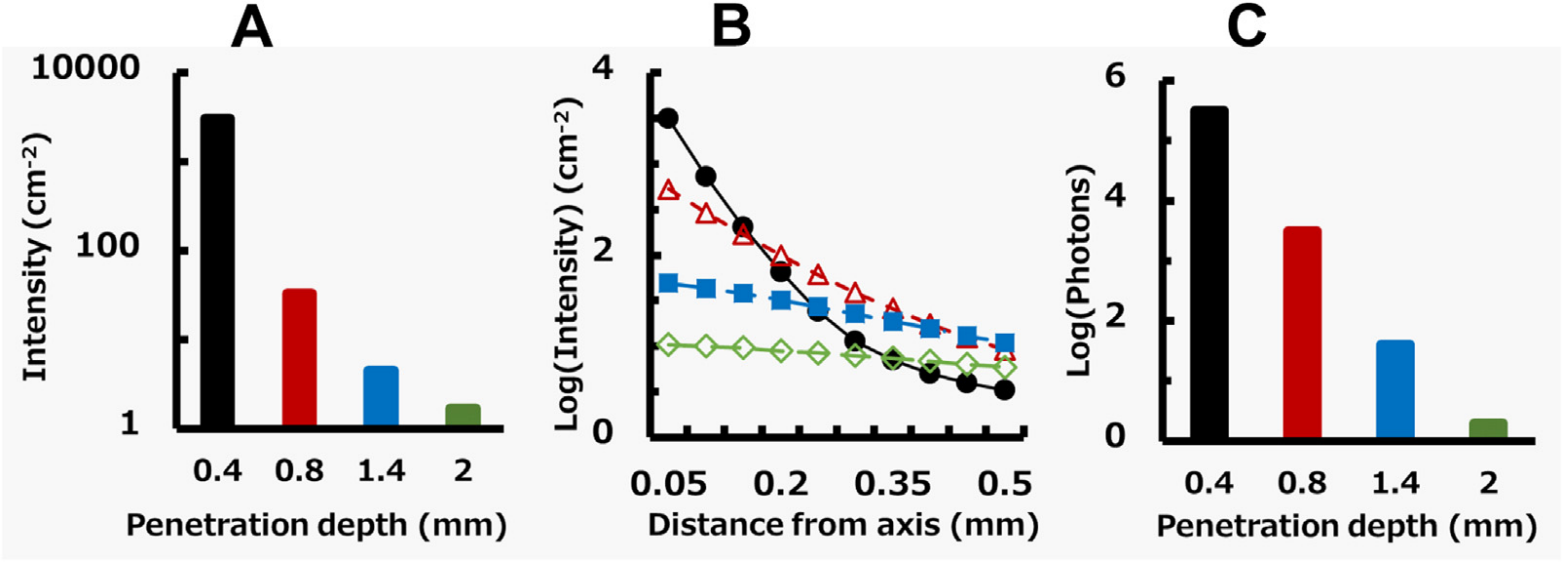
**Propagation properties of excitation and emission photons from the different depth, 0.2 (black), 0.6 (red), 1.2 (blue), 1.8 mm (green) from the skull surface.** (A) Excitation efficiency in different depths from the skull surface. (B) Relationship between emission intensity and distance from axis. Distance from axis denotes the bin range, namely 0.05 means 0-0.05 mm section. (C) Number of photons emitted from different depth.

Next, we examined the excitation efficiency of quantum dots at different depths from the skull surface. Fig. 3A shows the summation of photon weights per unit area on the light axis, i.e., intensity, at 0.4, 0.8, 1.4, and 2.0 mm depths. The intensity at 0.4 mm depth corresponding to the cortical surface was 3100 cm^−2^ and was the strongest among all depths. Deeper quantum dots got, the weaker the intensity became. The intensity at 0.8 mm depth equivalent to a 0.4 mm depth from the cortical surface was 33 cm^−2^ which was 1.1% of the intensity at 0.4 mm depth. The intensity at 1.4 mm and 2.0 mm depths was 0.15% and 0.054%, respectively. Thus, strong light scattering due to tissues resulted in a decrease in intensity in deeper tissues. Furthermore, it would affect the distribution of photons apart from the light axis.

Finally, we examined emission properties when 10^6^ photons with an initial weight of 1 were used as a point source in simulation at 0.4 mm depth, and compared them with those with weights of 0.011 at 0.8 mm depth, 0.0015 at 1.4 mm depth, and 0.0005 in 2.0 mm depth depending on the excitation efficiency shown in Fig. 3A. Fig. 3B shows the relationship between the intensity detected at equal intervals 0.05 mm apart from the light axis and distance. At 0.4 mm depth, the intensity of 0.05–0.1 mm section was <22% of that of 0–0.5 mm section. There were almost no photons with a distance farther than that of 0.1–0.15 mm section (<2%). At 0.8 mm depth, the intensity of 0.05–0.1 mm section was 55% of that of 0–0.05 mm section and the intensity of 0.1–0.15 mm section was 32%. The deeper the quantum dots got, the more widely emission photons spread. At 2.0 mm depth, the intensity of 0.45–0.5 mm section was >57% of that of 0–0.05 mm section. The relationship shown in Fig. 3B suggests that blur images were obtained due to deeper quantum dots. The quality of blur images can be improved using near-ballistic emission photons of which trajectories within a tissue were only partially deflected by scattering. The near-ballistic photons emitted at 0.4 mm depth were the most among the photons emitted at the four depths. The numerical aperture of the epifluorescence macro zoom microscope used in our previous study [5,13] was 0.25, indicating that the detection angle ranged between 0 and 15 degrees. As shown in Fig. 3C, when the photons near on-axis coaxial fluorescence within 15 degrees were collected using the optical system of the microscope, 3.1 × 10^5^ fluorescence photons were emitted from 0 to 0.4 mm depth. As the photons emitted from deeper points were counted, the number of photons decreased remarkably; 3.1 × 10^3^ at 0.8 mm depth, 40 at 1.4 mm, and 2 at 2.0 mm. Furthermore, similar to excitation, the deeper the quantum dots got, the more widely fluorescence photons spread (data not shown).

The use of this optical system facilitated preferential collection of these near-ballistic fluorescence photons relative to the diffusely scattered photons. Therefore, the fluorescence photons at 0.4 mm depth permitted brighter and clearer imaging of the vascular system near the cortical surface using this optical system, unlike photons emitted at 0.8, 1.4, and 2.0 mm depth. In contrast to the photons emitted in the visible region, the near-ballistic photons emitted in NIR-II tended to survive because photons that traveled deeper due to tissue scattering were absorbed by water, which can improve image quality. The depth of field of the microscope used in the present study was 0.3 mm when it was calculated using wavelength, numerical aperture, and magnification, according to the Berek equation [14]. This value is the distance that should be used to maintain a desired amount of image quality at a specified contrast without refocusing. In other words, the changes in image quality cannot be determined within the range of the depth of field. Furthermore, because scattering due to the scalp and skull was an obstacle to fine focusing, image quality was not very sensitive to the focal plane. Fluorescence was not observed from the layer shallower than the cortex. Although limited fluorescence may occur from the layer deeper than cortex surface, Monte Carlo simulation indicated that such photons were hardly detected by the microscope. Therefore, focusing at a depth of 0.4 mm from the scalp surface was practically suitable for the second near infrared neuroimaging of hairless mice with the epifluorescence macro zoom microscope.

So far, in a simple approach for nTOVI, the standard near-infrared fluorescent dye used was excited at a wavelength of 720 nm in NIR-I and had fluorescence spectra with the emission maximum at 780 nm in NIR-I. The preliminary treatment such as scalp shaving just before imaging facilitated non-invasive cerebrovascular observation. The quantum dots used in the present study were excited at a wavelength of 785 nm in NIR-I and emitted fluorescence at a wavelength of 1100 nm in NIR-II. For noninvasive imaging of a cerebrovascular structure, the use of hairless mice was feasible by injecting QD1100 under general anesthesia. As suggested by Monte Carlo simulation, the cerebrovascular structure was observed by setting focal plane 0.4 mm below the scalp surface.　

## Declaration of Competing Interest

We have no conflict of interest with any company or commercial organization.

## Figures and Tables

**Figure:**
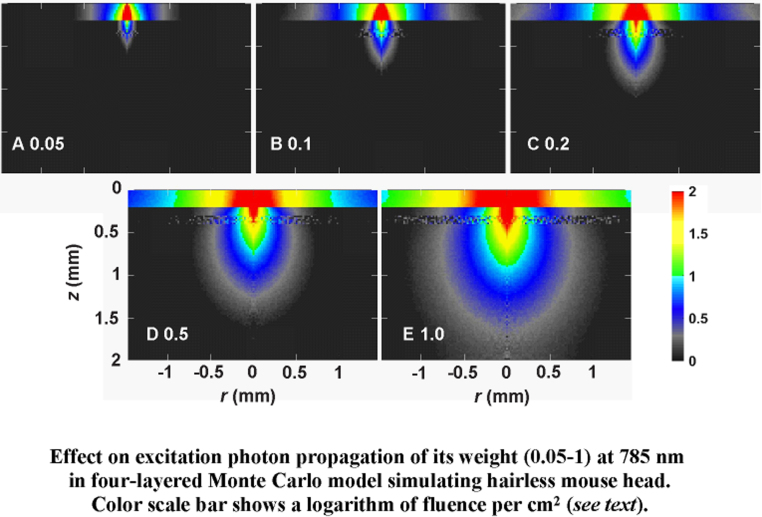


**Figure:**